# Introduction: Anti-inflammatory Treatment of Childhood Asthma: Cromoglycate and Nedocromil as Non-steroidal Alternatives?

**DOI:** 10.1155/S0962935194000670

**Published:** 1994

**Authors:** Johar C. de Jongste

**Affiliations:** 1 Department of Paediatrics Division of Paediatric Respiratory Medicine Erasmus University and University Hospital/ Sophia Children's Hospital Dr. Molewaterplein 60 Rotterdam 3015 GJ The Netherlands


Mediators of Inflammation 3, S5 (1994)

MOISU942

### Introduction

### Anti-inflammatory treatment of childhood asthma: cromoglycate and nedocromil as non-steroidal alternatives?

Since asthma has been recognized as a chronic inflammatory disorder of the airways, anti-inflammatory drugs and, in particular, corticosteroids have become first-line treatment.[1,2] Concern remains on the potential side-effects of inhaled corticosteroids especially in children.[3] Inhaled steroids have an excellent safety profile, and are widely used in paediatric asthma treatment.[4] Their long-term use in young children is generally thought to be both safe and effective, but small systemic effects can be demonstrated even with moderate doses.[5,6] These effects include the short-term inhibition of growth of long bones, and the suppression of basal cortisol secretion levels. Apart from these, there are risks of local side-effects including hoarseness and thrush. It is, however, generally felt that the benefits of steroids in asthmatic children greatly outweigh the small potential risks.

Despite the low toxicity of inhaled steroids, the availability of non-steroidal anti-inflammatory medication for treating asthmatic children is highly desirable because of the relatively high numbers of asthmatic children requiring maintenance treatment, and the long duration of anti-inflammatory asthma treatment. Two effective non-steroidal anti-asthma drugs with anti-inflammatory activities are presently available: cromoglycate and nedocromil. Of these cromoglycate has been used for many years and has proven to be both effective and extremely safe. Cromoglycate has been advocated as a first-choice drug for moderate paediatric asthma where maintenance treatment is required.[1] Much less data are available on nedocromil, a novel pyranoquinoline dicarboxylic acid with a broad spectrum of anti-inflammatory actions.[7] Nedocromil is more potent than cromoglycate, but it has been difficult to prove its superiority to cromoglycate *in vivo*.[8] Data on the effect of nedocromil in children with asthma are scanty, and tend to confirm its effectiveness in moderate asthma. The data presently available do not permit a clear positioning of the drug in clinical paediatric practice. Because nedocromil is one of the few drugs without steroid side-effects which can be applied in childhood asthma, and because it has anti-inflammatory effects, it deserves thorough study.

The present supplement issue of *Mediators of Inflammation* contains a selection of papers and abstracts on cromoglycate and nedocromil in childhood asthma from a recent symposium, where both clinical and laboratory studies on nedocromil were reported in order to get a better understanding of their mechanisms of action, especially in children. The editors think that publishing these original data may help in the further positioning of these drugs in the treatment of childhood asthma, an important and increasing disease with a major impact on childhood health.

**Johan C. de Jongste**
Department of Paediatrics,
Division of Paediatric Respiratory Medicine,
Erasmus University and University Hospital/
Sophia Children's Hospital,
Dr. Molewaterplein 60,
3015 GJ Rotterdam,
The Netherlands

### References

1. Warner JO. Asthma: a follow-up statement from an international pediatric asthma consensus group. *Arch Dis Childhood* 1992; **67**: 240–248.
2. Barnes PJ, Pedersen S. Efficacy and safety of inhaled corticosteroids in asthma. *Am Rev Respir Dis* 1993; **148**: S1–S26.
3. Kamada AK, Parks DP, Szefler SJ. Inhaled glucocorticoid therapy in children: how much is safe? *Pediatric Pulmonol* 1992; **12**: 71–72.
4. Kerrebijn KF. Use of topical corticosteroids in the treatment of childhood asthma. *Am Rev Respir Dis* 1990; **141**: S77–S81.
5. Pedersen S. Safety aspects of corticosteroids in children. *Eur Respir Rev* 1994; **4**: 33–43.
6. König P. The risks and benefits of inhaled corticosteroids. *Eur Respir Rev* 1993; **3**: 15, 501–510.
7. Thomson N. Nedocromil sodium: an overview. *Respir Med* 1989; **83**: 269–276.
8. Edwards AM, Stevens MT. The clinical efficacy of inhaled nedocromil sodium (Tilade) in the treatment of asthma. *Eur Respir J* 1993; **6**: 35–41.

© 1994 Rapid Communications of Oxford Ltd
